# Supplementary material for: Identification of photoperiod-induced specific miRNAs in the adrenal glands of Sunite sheep (Ovis aries)
Source: Front Vet Sci. 2022 Jul 22;9:888207. doi: 10.3389/fvets.2022.888207 (PMC9354845; doi:10.3389/fvets.2022.888207)
Supplement: Supplementary file 1 [file Data_Sheet_1.ZIP › Supplementary file/miRDeep2 file.zip!/miRDeep2 file/Know_Structure/LP42A1/oar-let-7g.pdf]

miRBase precursor : oar-let-7g
Total read count : 800923
oar-let-7g read count : 800904
remaining reads : 19

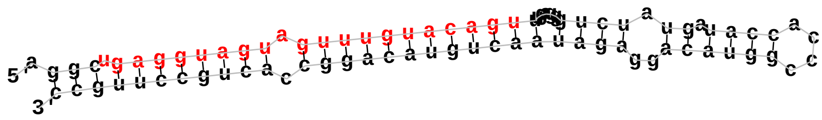

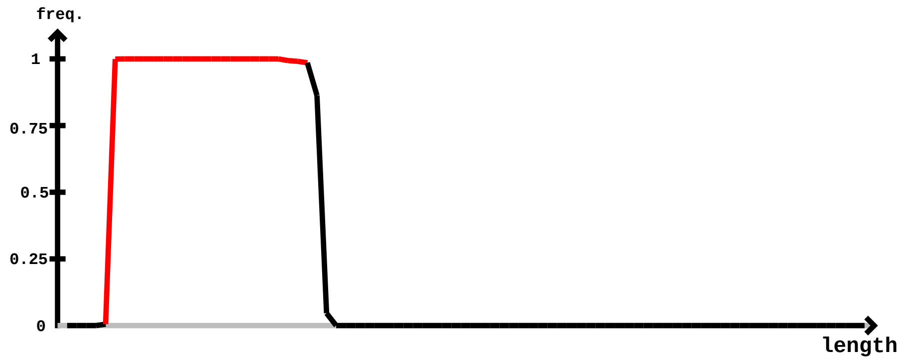

oar-let-7g

5'- aggcugagguaguaguuuguacaguuugagggucuaugauaccacccgguacaggagauaacuguacaggccacugccuugcc -3'	exp		
.(((.((((((((.((((((((((((.....((((.((.((((....))))))..)))))))))))))))).)))))))))))	reads	mm	sample
...cugagguaguaguuugu...	1	0	seq
...cugagguaguaguuugua..	1	0	seq
...cugagguaguaguuuguac...	30	0	seq
...cugagguaguaguuuguaca..	12	0	seq
...cugagguaguaguuuguacag...	32	0	seq
...cugagguaguaguuuguacagu..	793	0	seq
...cugagguaguaguuuguacaguu...	3109	0	seq
...cugagguaguaguuuguacaguuu..	161	0	seq
....ugagguaguaguuugua..	135	0	seq
....ugUgguaguaguuugua..	2	1	seq
....ugagguagCaguuuguac...	7	1	seq
....ugagguaguaguuuguac...	4971	0	seq
....ugagguaguaguuuguaca..	2194	0	seq
....ugagguaguaguuuguacag...	4177	0	seq
....ugagguaguaguuuguacagu..	97211	0	seq
....ugagguaguaguuuguacaguu...	650753	0	seq
....ugagguaguaguuuguacaguuu..	36822	0	seq
....ugagguaguaguuuguacaguuug...	21	0	seq
....ugagguaguaguuuguacaguuuga..	1	0	seq
....ugagguaguaguuuguacaguuugagggu..	1	0	seq
.....gagguaguaguuuguaca..	1	0	seq
.....gagguaguaguuuguacag...	2	0	seq
.....gagguaguaguuuguacagu..	41	0	seq
.....gagguaguaguuuguacaguu...	395	0	seq
.....gagguaguaguuuguacaguuu..	27	0	seq
......agguaguaguuuguacagu..	2	0	seq
......agguaguaguuuguacaguu...	3	0	seq
....cuguacaggccacugccuug..	1	0	seq
....cuguacaggccacugccuugc.	11	0	seq
....cuguacaggccacugccuugcc	6	0	seq
